# Sequential Targeted Temperature Management: Case Report and Literature Review

**DOI:** 10.7759/cureus.5012

**Published:** 2019-06-26

**Authors:** Sebastian Casillas, Joseph Varon, Salim Surani

**Affiliations:** 1 Research, Dorrington Medical Associates, Houston, USA; 2 Critical Care, University of Texas Health Science Center and United General Hospital, Houston, USA; 3 Internal Medicine, Texas A&M Health Science Center, Temple, USA

**Keywords:** targeted temperature management, hypothermia, cardiac arrest, radiation chondronecrosis, compromised airway

## Abstract

We present the case of a 59-year-old gentleman with a history of nonmetastatic tonsillar malignancy and radiation chondronecrosis, who underwent targeted temperature management (TTM) in a sequential manner. The first time the patient underwent therapeutic cooling occurred after he developed a respiratory arrest followed by a cardiac arrest and prolonged hypoxemia after a diagnostic laryngoscopy. The patient was kept at 32°C for 24 hours, and 48 hours after rewarming woke up neurologically intact. However, six hours post-extubation, he suffered upper airway obstruction, followed by a prolonged cardiac arrest. Return of spontaneous circulation on this second episode was achieved after 45 minutes of resuscitation maneuvers. The patient was cooled again and kept at 32°C for 48 hours. Five days later, the patient recovered, with an intact neurologically function. This case stands out the importance of sequential TTM after repeat cardiac arrests with a return of spontaneous circulation (ROSC), demonstrating this process as a neuroprotective way of treatment.

## Introduction

Targeted temperature management (TTM), previously known as therapeutic hypothermia (TH), has been used for at least 100 years for patients of sudden cardiac arrest (CA) [1-3]. Several studies have shown that TTM has a reasonable safety profile, and that is efficacious as a neuroprotective measure [4-5]. Targeted temperature management is commonly used for 24-72 hours at temperatures that range between 32-36°C [5-6]. Sequential TTM has rarely been reported [7]. We recently had one such case.

## Case presentation

**Figure 1:**
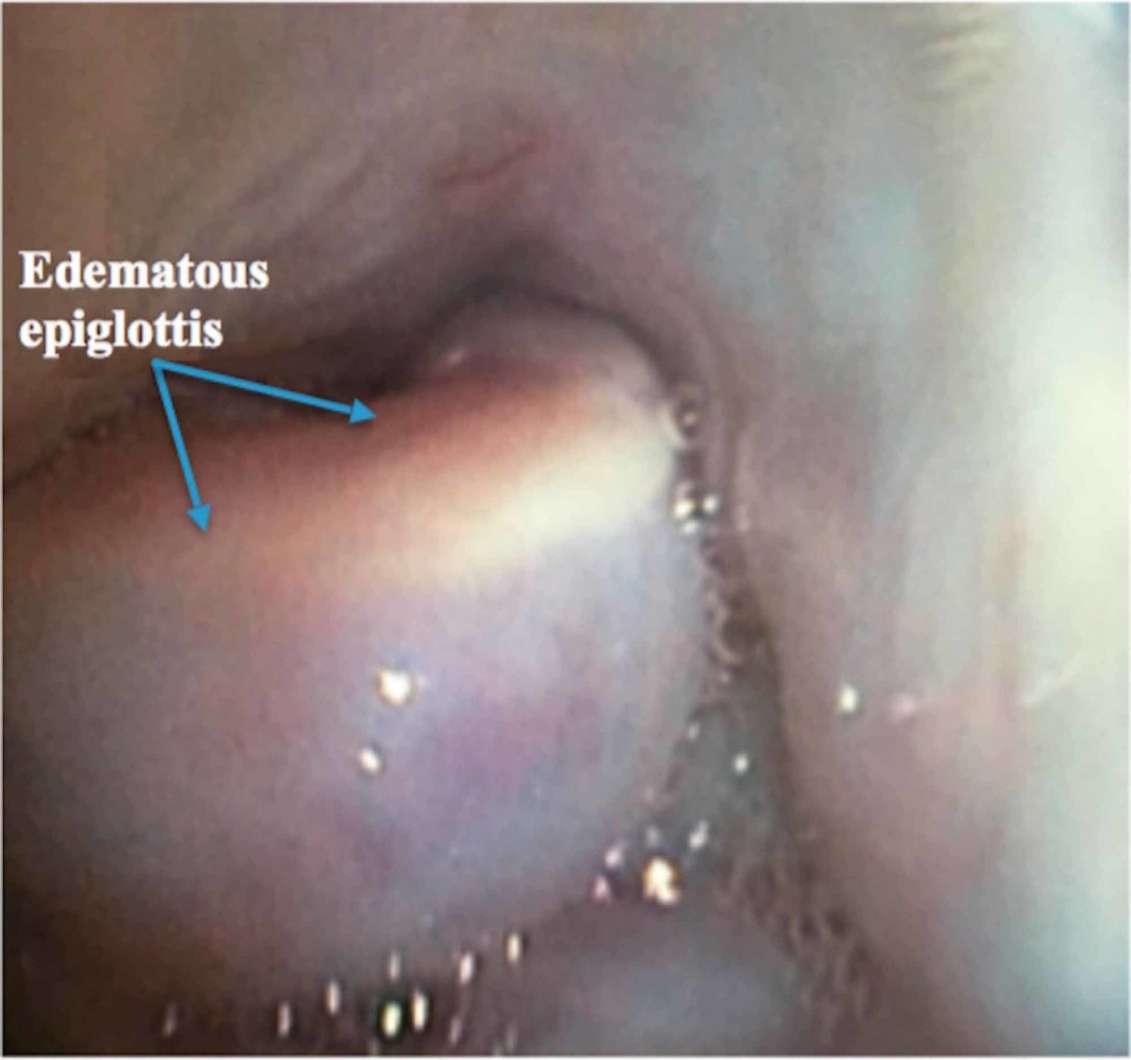
Severely edematous epiglottis obstructing the view of the vocal cords

A 59-year-old man with a history of nonmetastatic tonsillar malignancy and post radiation stridor, was referred to our service for evaluation of his upper airway. He complained of decreased cervical range of motion and severe dysphagia. An elective direct upper laryngoscopy was performed under conscious sedation. He was found to have a markedly edematous epiglottis (see Figure 1).

Within minutes, the patient’s oxygen saturation dropped to 60% while breathing supplemental oxygen at 100% via a non-rebreather mask. Bag-valve ventilation was attempted with no success. His heart rate dropped to 30 beats per minute. Several attempts of intubation were unsuccessful due to the severe narrowing of his upper airway and the inability to move his neck. He developed a respiratory arrest that was followed by pulseless electrical activity. Laryngeal mask airway placement was also unsuccessful due to small oral opening. As the patient had significant fibrosis of the anterior neck, cricothyrotomy was not feasible. Cardiopulmonary resuscitation (CPR) was started and within 20 minutes, (after successful intubation was accomplished with 5 F endotracheal tube) return of spontaneous circulation (ROSC) was obtained. Relevant laboratory data are detailed in Table 1. 

**Table 1:** Laboratory findings

Laboratory results		
Comprehensive Metabolic Panel (CMP)		
	Results	Normal Range
Sodium	138 mmol/L	135-146 mmol/L
Potassium	4.1 mmol/L	3.5-5.3 mmol/L
Chloride	107 mmol/L	98-110 mmol/L
Carbon dioxide	24 mmol/L	20-31 mmol/L
Calcium	8.4 mg/dL	8.6-10.3 mg/dL
Special laboratory request		
	Results	Normal Range
Lactic acid	1.8 mmol/L	0.5-2.2 mmol/L
Arterial Blood Gas (ABG)		
	Results	Normal Range
pH	7.44	7.35-7.45
pCO_2_	32 mmHg	35-45 mmHg
pO_2_	159 mmHg	80-100 mmHg
HCO_3_	22 mEq/L	22-26 mEq/L

The patient underwent TTM with ice packs and cooling blankets until his core temperature reached 32°C. This temperature was maintained for 24 hours. The patient was rewarmed over 24 hours to the temperature of 36.5°C. Mean arterial pressure (MAP), heart rate, temperature, and Glasgow coma scale (GCS) were monitored at the same time. Within minutes after discontinuation of the sedative agents, the patient began following commands, had a good gag reflex and deep tendon reflexes. The patient was extubated after having a good leak on deflation of the endotracheal tube cuff. He was able to vocalize and had no stridor. Six hours post-extubation, the patient complained of dyspnea and started having oxygen desaturation. On exam, no stridor was present and attempt of non-invasive ventilation failed.

Anesthesiology attempted intubation several times. During this process, the patient once again developed cardiopulmonary arrest. Again, CPR was initiated as per standard American Heart Association (AHA) and Advanced Cardiac Life Support (ACLS) protocol. The patient was successfully intubated again with difficulty with 5 French endotracheal tube. After 45 minutes of CPR, ROSC was achieved one more time. The patient’s GCS was 3/15. At that point, a decision was made to reinitiate TTM once again. The patient was cooled to 32°C for 24 hours. He was then rewarmed over a 24-hour period. However, this time, once sedative agents were weaned off, the patient remained unresponsive. His pupils were equal, round and reactive. He had no response to painful or auditory stimuli. He remained neurologically unchanged for 48 hours, and on the third day, he began opening his eyes upon auditory stimuli and withdrawing upon painful stimuli. On the fifth day post rewarming, he was fully awake and alert. He underwent permanent tracheostomy placement and gastrostomy tube placement and was weaned from assisted ventilation two days later. He was discharged to home. On three months follow up, the patient had no neurological deficits and had undergone an upper airway and esophageal reconstruction; the tracheostomy and gastrostomy were removed.

## Discussion

Current practice guidelines recommend TTM in CA patients successfully resuscitated with persistent coma, as a Class I recommendation if the arrest had an initial ventricular tachycardia or ventricular fibrillation, and Class IIb recommendation from other non-shockable rhythms [8]. The European Resuscitation Council guidelines for resuscitation recommend TTM for all comatose survivors of CA regardless of initial rhythm, although the guidelines recognize a lower level of evidence for TTM in patients with a CA from non-shockable rhythms [9].

The cornerstone of management in patients that suffer a CA with ROSC is to attempt to provide the best possible neurological prognosis. Targeted temperature management can improve neurological recovery of these patients [10]. Although not completely clear, the alleged neuroprotective effects of TTM have several molecular and cellular mechanisms. For example, in TTM a reduction of the extracellular levels of excitatory neurotransmitters occurs [11]. Cooling also decreases the activation of the intrinsic and extrinsic apoptotic pathways, as well as, mitigates reperfusion injury, by protecting the lipoprotein membrane integrity and preventing apoptosis [2,11].

Clinically, the data supports TTM in the management of these patients. One cohort study conducted by Chan et al. compared TTM and non-hypothermia-treated patients after in-hospital CA, showing a lower likelihood of survival after one time TTM (27.4% vs 29.2%), and a lower favorable neurological survival (17.0% vs 20.5%) [12]. Perman and associates also showed not only a neurological improvement but also a greater survival to hospital discharge in patients who underwent TTM than the ones who did not receive it (17.6% vs. 28.9%) [13].

A large meta-analysis comparing survival and neurological recovery at discharge in groups that underwent TTM against groups that did not receive it, revealed that patients in TTM group were more likely to be discharged with none or minimal neurological sequelae (risk ratio 1.68 and *p* value .006); it is worth mentioning that in this study, the patients had follow-up for six months and the TTM group had better neurological recovery (risk ratio, 1.44 and *p* value .009) [14].

The exact duration and depth of TTM remain a matter of controversy [15-16]. To our knowledge, there have been no randomized clinical trials specifically addressing consecutive TTM in in-hospital CA. Our case depicts that consecutive TTM is safe and can be efficacious. Indeed, in our case, the patient tolerated more than one TTM “session” reaching the target temperature without any complications. We had previously described another case where a patient underwent TTM twice successfully [17]. However, the present case is different in the duration of the arrest, the pre-existing conditions, and the excellent outcome that our patient had.

## Conclusions

Sequential TTM can be brain-saving and should be considered in patients that have a repeat CA with ROSC and coma. There is clearly a need to conduct a longitudinal study for sequential TTM in patients who have more than one CA.
